# Correction to “A Case of *CSNK2A1* Gene Variant Causing Okur‐Chung Syndrome and Analysis of the Clinical Phenotypic Spectrum”

**DOI:** 10.1002/mgg3.70280

**Published:** 2026-08-12

Li, X. , S. Wang , X. Liu , et al. 2025. “A Case of *CSNK2A1* Gene Variant Causing Okur‐Chung Syndrome and Analysis of the Clinical Phenotypic Spectrum.” Molecular Genetics & Genomic Medicine 13, no. 12: e70166. 10.1002/mgg3.70166.41395761 PMC12703824

In the original article, the authors inadvertently omitted a citation to an earlier Chinese‐language publication reporting the initial clinical description of the proband and the primary genetic findings (corresponding to Figures 1–4). The present MGGM article expands upon that preliminary report by including additional analyses of 65 retrospective cases and new quantitative genotype–phenotype analyses (Tables 2–4). The omitted reference is:

Li, X., S. Wang, C. Li, et al. 2025. “A Case of Okur‐Chung Syndrome Caused by CSNK2A1 Gene Mutation and Analysis of Clinical Manifestations.” *International Journal of Genetics (in Chinese)* 48, no. 4: 326–333. DOI: https://doi.org/10.3760/cma.j.cn231536‐20250113‐00002.

We apologise for this error.

